# Mediterranean diet impact on plasma proteins: biomarker networks linking nutrition to the modulation of inflammation and oxidative stress

**DOI:** 10.3389/fnut.2026.1904329

**Published:** 2026-07-28

**Authors:** Fátima Cardoso, Eduardo Costa-Camilo, Isabel Duarte, Graça P. Carvalho, Rita G. Sobral, João M. G. C. F. de Almeida, Carla Pinheiro

**Affiliations:** 1 UCIBIO Applied Molecular Biosciences Unit, Department of Life Sciences, NOVA School of Science and Technology, Universidade NOVA de Lisboa, Caparica, Portugal; 2 Associate Laboratory i4HB Institute for Health and Bioeconomy, NOVA School of Science and Technology, Universidade NOVA de Lisboa, Caparica, Portugal; 3 Instituto Nacional de Investigação Agrária e Veterinária (INIAV), Elvas, Portugal; 4 GeoBioTec, NOVA School of Science and Technology (NOVA FCT), Universidade NOVA de Lisboa, Caparica, Portugal; 5 Bioscience School of Elvas, Portalegre Polytechnic University, Elvas, Portugal; 6 Research Center for Endogenous Resource Valorization (VALORIZA), Portalegre, Portugal

**Keywords:** aging, bioactive compounds, molecular pathways, plasma protein biomarkers, therapeutical potential

## Abstract

The Mediterranean diet (MedDiet) is widely recognized for its role in preventing chronic and age-related diseases, largely attributed to its richness in plant-based foods and bioactive compounds. To explore the underlying molecular mechanisms, two systematic literature searches were conducted using PubMed and Web of Science. The first assessed the evidence on the health effects of key MedDiet components and the second focused on plasma biomarkers of inflammation and oxidative stress. Protein-protein interaction network analysis revealed a tightly connected metabolic network integrating inflammatory mediators and metabolic regulators. Central nodes included key inflammatory markers such as interleukin-6, tumor necrosis factor and C-reactive protein (CRP), alongside proteins involved in lipid metabolism and metabolic homeostasis. Enrichment analysis highlighted pathways related to acute inflammatory response, lipid localization and storage and plasma lipoprotein particle clearance, supporting a role in regulation of immune and metabolic processes. An additional network integrating cardiovascular aging biomarkers suggested a partial separation between systemic inflammatory pathways and markers of myocardial stress and injury, with CRP emerging as a potential link between these processes. Overall, these findings indicate that the MedDiet may exert its health benefits through systems-level modulation of interconnected metabolic pathways, contributing to the regulation of inflammation, lipid metabolism and cardiovascular function.

## 1 The Mediterranean diet

The Mediterranean diet (MedDiet) is globally recognized for its significant health benefits and balanced nutritional profile (1–5). It typically provides approximately 50% of daily energy from carbohydrates, 30% from fats and 20% from proteins, while emphasizing the use of fresh, seasonal and minimally processed ingredients (6). Its culinary practices are rooted in simplicity and quality, highlighting local products and traditional cooking methods that prioritize both taste and nutritional value (7). In 2011, UNESCO acknowledged it as an Intangible Cultural Heritage of Humanity, initially recognizing Italy, Greece, Spain and Morocco (8). In 2013, this recognition was extended to include Cyprus, Croatia and Portugal (8). The diet is closely linked to lifestyle factors such as shared meals, physical activity and sleep patterns, making it more than just a way of eating.

The MedDiet is especially rich in phytochemicals, plant secondary metabolites that support plant defense, growth and pigmentation, that have bioactive properties and provide antioxidant and anti-inflammatory effects (5, 9). The concept of “nutraceuticals,” introduced in 1979, refers to the medicinal use of food-derived compounds (10). As the interest in functional foods grew, nutraceuticals became a promising link between nutrition and medicine, further supporting dietary models like the MedDiet in disease prevention (9, 11–14). This is particularly relevant in the context of the global rise of Non-Communicable Diseases (NCDs), including for example heart disease, stroke, cancer, diabetes and chronic respiratory illnesses (15, 16). They are the leading global cause of mortality, responsible for over 41 million deaths annually (more than 70% of all deaths) (17, 18). Of these, 15 million are pre-mature (ages 30–69), with 85% occurring in low- and middle-income countries (19, 20). In the European Union, NCDs account for 80% of the disease burden and are the main cause of avoidable pre-mature fatalities (21–23). A 2011 study linked health inequalities in the European Union to over 700,000 deaths and 33 million illnesses per year, representing 20% of healthcare and 15% of social security costs (24). Contributing factors include health literacy, access to care, economic status and national policies. In Portugal, by 2015–2016, over 20% of the population was obese and by 2017, 88% of all deaths were due to NCDs (24). These numbers reflect a wider trend observed across Europe, where lifestyle-related chronic diseases are rising, highlighting the urgent need for preventive strategies.

**Figure 1:**
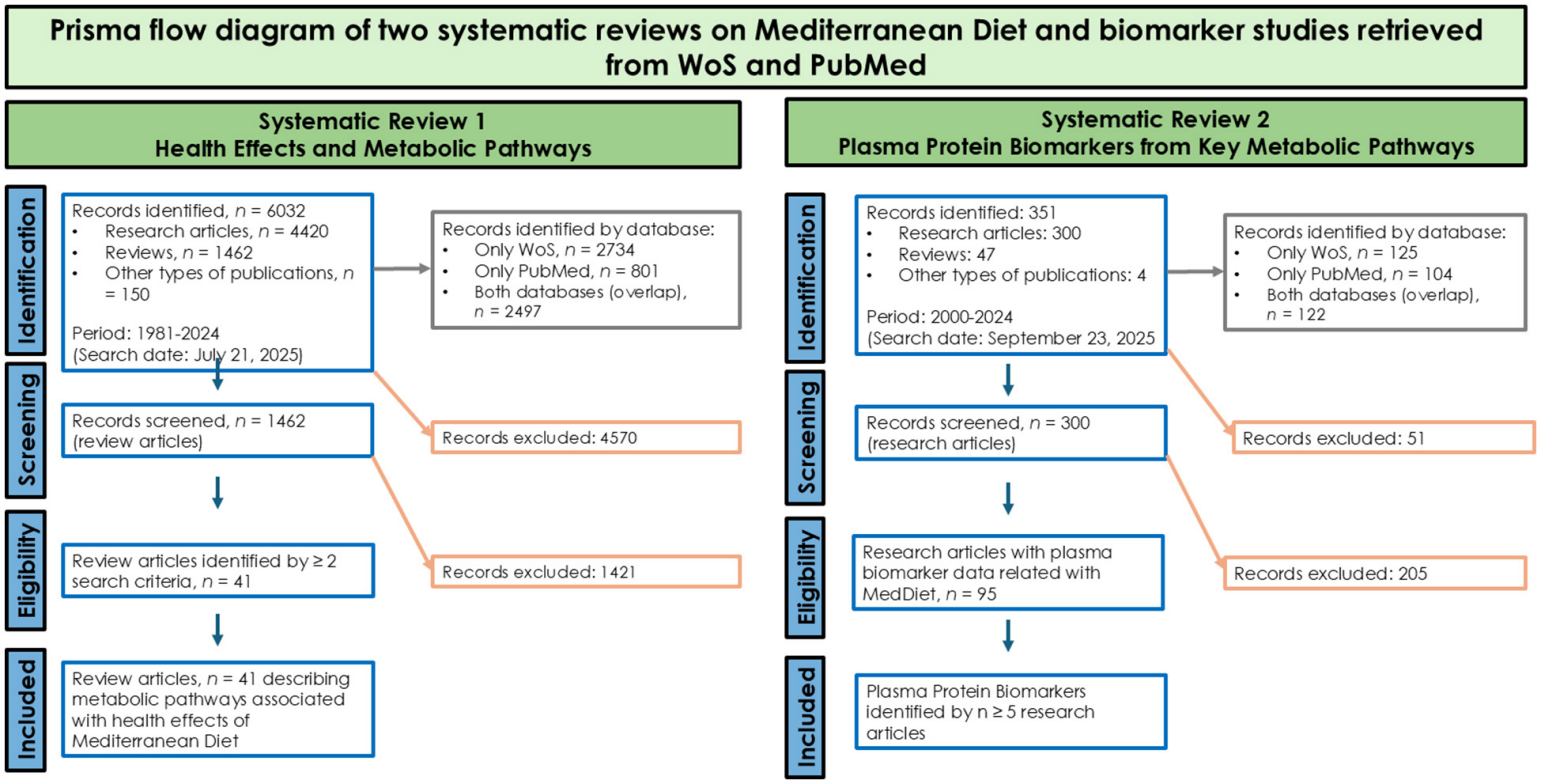
PRISMA diagram of the two systematic reviews on Mediterranean Diet and biomarker studies retrieved fromWoS and PubMed.

Considering the prevalence of plant-based foods in the MedDiet, a comprehensive literature review was conducted to capture evidence on the health effects of its key components. The search was performed using the Web of Science (WOS) and PubMed databases (as of 21 July 2025), combining “Mediterranean diet” with individual foods and food ingredients (Table 1) (25). The WOS search tool was restricted to the Science Citation Index Expanded database. Studies were selected according to pre-defined search and eligibility criteria (Table 1, Figure 1).

**Table 1:** Search terms used to identify major health benefits of the Mediterranean diet plant-based foods in WOS and PubMed.

Search topic	Number of publications	Number of publications just indexed in WOS	Number of publications just indexed in PubMed	Selected reviews
(Mediterranean diet) AND (olives and olive oil)	3,038	1,386	248	(75–81)
(Mediterranean diet) AND (wine consumption)	484	213	65	(82–85)
(Mediterranean diet) AND (herbs and spices)	51	20	4	(86–89)
(Mediterranean diet) AND (fruits and vegetables)	2,465	913	323	(67–70)
(Mediterranean diet) AND (whole grains)	688	375	59	(90–93)
(Mediterranean diet) AND (legumes)	970	294	130	(94–96)
(Mediterranean diet) AND (nuts and seeds)	162	60	36	(97, 98)
(Mediterranean diet) AND (fermented foods)	302	45	229	(99, 100)
(Mediterranean diet) AND (coffee)	261	136	21	(101–103)
(Mediterranean diet) AND (tea)	331	189	30	(104, 105)
(Mediterranean diet) AND (cocoa)	90	28	41	(65, 106–108)
Publications retrieved by:				
One search criterium	4,268			
Two search criteria	1,049			
Three search criteria	473			
Four search criteria	176			
Five to eight search criteria	66			

The number of publications per search string and database, as well as selected reviews are indicated.

“Publications” refers to all types of records present in the databases, including research articles, reviews, proceedings papers and meeting abstracts, book chapters, corrections, retractions and comments, letters and editorials published in English. The complete list of retrieved publications is provided as Supplementary Table S1.

The search returned a total of 6,032 publications, including 4,420 research articles, 1,462 reviews, and 150 from other categories (e.g., comments/letters/editorials), covering the period between 1981 and 2024. Some records were available in both WOS and PubMed (n = 2,497), while 2,734 were indexed only in WOS and 801 only in PubMed. In addition, 4,268 publications were retrieved exclusively by one search criterion, whereas 1,764 were identified by two or more search criteria. Following the application of the pre-defined eligibility criterion of being a review article retrieved by at least two of the search criteria listed in Table 1, 41 studies were selected for further analysis (Table 2).

**Table 2:** Major components of the Mediterranean diet and associated health benefits.

Foods/ingredients	Main components	Health benefits	Selected references
Olives and olive oil	Phenolic compounds (e.g., hydroxytyrosol, tyrosol, oleuropein, caffeic and coumaric acids)	Analgesic/symptom relief: analgesic, purgative Anti-inflammatory/immune modulation: anti-inflammatory, immunomodulatory Antimicrobial/antiviral effects: antimicrobial, antiviral Cellular protection: antioxidant, hepatoprotective Metabolic/cardiovascular effects: hypoglycemic, anti-atherogenic	(25, 75–80)
Wine	Phenolic compounds (e.g., resveratrol, tyrosol, quercetin, anthocyanins, flavonoids)	Anti-inflammatory/immune modulation: anti-inflammatory Antimicrobial/antiviral effects: antimicrobial Cellular protection: antioxidant Metabolic/cardiovascular effects: cardioprotective	(81–84)
Aromatic herbs and spices	Phenolic compounds (e.g., curcuminoids, rosmarinic acid), alkaloids (e.g., capsaicinoids, piperine), organosulfur compounds (allicin), glycosides (saponins)	Anti-inflammatory/immune modulation: anti-inflammatory Antimicrobial/infection control: antimicrobial Cardiovascular protection: cardioprotective, antihypertensive Cellular protection: antioxidant, hepatoprotective, neuroprotective support Metabolic/endocrine effects: antidiabetic, metabolic support	(85–89)
Fruits	Anthocyanins (e.g., cyanidin, delphinidin, malvidin)	Anticancer/disease-modifying effects: anticancer Anti-inflammatory/immune modulation: Cardiovascular protection: cardioprotective Cellular protection: antioxidant Neuroprotective effects: cognitive support	(67–70)
Vegetables	Phenolic compounds (e.g., oleuropein, catechin, quercetin, eugenol) and pigments (carotenoids)	Cellular protection: antioxidant Structural/systemic support: bone, muscle, digestive system, vision and microbiome health	
Whole grains	Phenolic acids (e.g., hydroxybenzoic and hydroxycinnamic acids)	Cellular protection: antioxidant	(90–93)
Legumes	Phenolic compounds (e.g., flavonoids, isoflavones, anthocyanins, tannins)	Anticancer/disease-modifying effects: anticancer Anti-inflammatory/enzyme modulation: anti-inflammatory, Immunomodulatory Cellular protection: antioxidant Metabolic/cardiovascular effects: antidiabetic, antihypertensive, cardioprotective	(94–96)
Nuts and seeds	Healthy fats, fiber, phenolic compounds, phytosterols, pigments and vitamins	Anticancer/disease-modifying effects: anticancer Anti-inflammatory/enzyme modulation: anti-inflammatory Cardiovascular protection: cardioprotective Cellular protection: antioxidant	(97–99)
Fermented foods	Phenolic compounds (e.g., caffeic acid, ferulic acid, gallic acid)	Cellular protection: antioxidant	(100, 101)
Coffee	Alkaloids (e.g., caffeine, trigonelline), chlorogenic acids, diterpenes	Anticancer/disease-modifying effects: anticancer Anti-inflammatory/enzyme modulation: anti-inflammatory Cardiovascular protection: antihypertensive Cellular protection: antioxidant Metabolic/endocrine effects: antidiabetic	(102–104)
Tea	Phenolic compounds (e.g., catechins)	Anticancer/disease-modifying effects: anticancer Anti-inflammatory/immune modulation: anti-inflammatory Antimicrobial/antiviral effects: bacteriostatic Cellular protection: antioxidant Metabolic/cardiovascular effects: lipid regulation	(105, 106)
Cocoa	Phenolic compounds n(e.g., catechins, flavonols, anthocyanins, procyanidins)	Anti-inflammatory/enzyme modulation: inhibits lipoxygenase, reduces inflammation Cellular protection: antioxidant, prevents lipid peroxidation Cognitive/neurological effects: improves cognitive function	(26, 65, 107, 108)

The complete list of retrieved publications is available as Supplementary Table S1.

Cellular protection and anti-inflammatory/immune modulation are the most referred functional effects. Oxidative stress and chronic low-grade inflammation are central drivers of both aging and NCDs, including cardiovascular disease, type 2 diabetes, cancer, and neurodegeneration (9, 12–14, 26, 27). These mechanisms involve dysregulated cytokine production, immune activation, and imbalances in reactive oxygen species. Evidence shows that the MedDiet helps reduce inflammation by lowering white blood cell counts, downregulating immune pathways, and decreasing markers such as C-reactive protein (CRP) and tumor necrosis factor-alpha (TNF) (26, 27).

At the molecular level, the influence of diet on inflammation and oxidative stress can be assessed through plasma biomarkers (12, 28). Plasma reflects nutrient bioavailability and systemic metabolic changes, providing an objective measure of dietary adherence and physiological responses while overcoming the limitations of self-reported data. To investigate these associations, we conducted a second systematic literature search to identify studies examining the relationship between MedDiet adherence and plasma biomarkers of inflammation and oxidative stress (7). The search conducted using WOS and PubMed (as of 23 September 2025; Table 3, Figure 1), returned a total of 351 publications (300 research articles and 47 reviews) published between 2000 and 2024, including 172 retrieved exclusively by one search criterion. After restricting the analysis to original research articles investigating plasma biomarkers (proteins or metabolites associated with the MedDiet), 95 relevant studies were included (Supplementary Table S3). Within these, only biomarkers reported in at least three studies were retained, aiming to broadly characterize the molecular profile associated with the MedDiet and capture a wider range of potentially relevant biomarkers. Studies not focusing on the MedDiet, lacking relevant plasma biomarker data, or not assessing the effects of diet on plasma biomarkers were excluded (*n* = 205).

**Table 3:** Summary of studies investigating Mediterranean diet–associated plasma biomarkers related to inflammation and aging, identified based on the search criteria.

Search topic	Number of publications	Number of publications just indexed in WOS	Number of publications just indexed in PubMed
(Mediterranean diet) AND (plasma biomarkers)	349	126	104
(Mediterranean diet) AND (plasma biomarkers) AND (inflammatory response)	113	84	18
(Mediterranean diet) AND (plasma biomarkers) AND (aging)	25	9	7
(Mediterranean diet) AND (plasma biomarkers) AND (cellular aging)	6	3	3
(Mediterranean diet) AND (plasma biomarkers) AND (oxidative stress)	89	35	30
Publications retrieved by:				
One search criterium	172		
Two search criteria	129		
Three search criteria	48		
Four search criteria	2		

“Publications” refers to all types of records present in the databases and includes research articles and clinical reports, reviews, proceedings papers, book chapters, corrections, retractions and comments, letters and editorials published in English. The complete list of retrieved publications is provided as Supplementary Table S2.

The comprehensive overview of the 95 publications (Supplementary Table S4) allowed the identification of a subset of biomarkers associated with adherence to the MedDiet, along with their corresponding physiological effects or pathological conditions resulting from their dysregulation (14, 29–61). Collectively, these biomarkers reflect essential biological processes that will be detailed in the following sections.

## 2 Exploring plasma biomarkers linking the Mediterranean diet, inflammation and aging

Adherence to the MedDiet is associated with lower levels of pro-inflammatory markers, including CRP, several interleukins (IL1B, IL6, IL8, IL12B, IL15, IL18), TNF, interferon-gamma (IFNG) (29, 38–41, 43, 44, 48, 59). It also modulates immune-related proteins such as chemokines (CCL2, CCL5) and TNF receptors (62). Also reported were the increase levels of anti-inflammatory lipid mediators such as resolvin D1 and E1 (63). MedDiet may also support brain health and neuroprotection by influencing markers such as leukocyte immunoglobulin-like receptors, fibroblast growth factors (stanniocalcin-1 and proteins associated with neurodegeneration, including hepatocyte growth factor) (62).

### 2.1 Oxidative stress and antioxidant defense

The MedDiet provides nutrients with antioxidant properties, including vitamins, carotenoids, phenolic compounds and polyunsaturated fatty acids, that can contribute to the reduction of the oxidative stress. MedDiet adherence is reflected by lower levels of oxidized LDL and MDA (malondialdehyde, an oxidative damage marker), along with increased levels of IL10 and polyunsaturated fatty acids (eicosapentaenoic acid and docosahexaenoic acid) that are antioxidant biomarkers (37, 55, 56). Enzymatic defenses are also enhanced, with decreased pro-oxidant enzymes and increased antioxidant enzymes, leading to improved oxidative balance (63, 64). These changes are associated with reduced risks of atherosclerosis, diabetes, cancer and mortality (37, 55, 56, 63).

Complementary evidence supporting the antioxidant potential of MedDiet is provided by studies examining the effects of Mediterranean-style foods on plasma redox status. Within our research group, in an exploratory study, consumption of Mediterranean-style soups was associated with modulation of antioxidant profiles and reduced variability in redox-related markers, particularly in older individuals (65). The MedDiet has also been associated with activation of the nuclear factor erythroid 2–related factor 2 pathway, a key regulator of the cellular antioxidant response (63, 66). The activation of this regulator promotes the expression of antioxidant and cytoprotective enzymes, thereby reducing oxidative stress, which is closely linked to chronic inflammation and cardiometabolic dysfunction (63, 66). These findings are consistent with age-related oxidative stress and suggest that Mediterranean dietary patterns may contribute to the stabilization of systemic redox balance (66).

### 2.2 Metabolic and vascular effects

The search also retrieved biomarkers related to lipid and glucose metabolism, as well as endothelial and vascular function. These are interconnected processes as inflammatory mediators such as cytokines, oxidized lipoproteins, and bacterial endotoxins can activate NF-κB signaling, up-regulate adhesion molecules and chemokines, and reduce nitric oxide availability, contributing to endothelial dysfunction and early vascular damage (48, 49). MedDiet further supports endothelial and vascular health by reducing circulating adhesion molecules (SELE, VCAM1, ICAM1) and lower pro-atherogenic metabolites (TMAO, L-carnitine, choline), which collectively protect against vascular inflammation and cardiovascular disease (48, 49, 63). Age-related oxidative stress, driven by mitochondrial damage and endothelial nitric oxide synthase uncoupling, further promotes a pro-inflammatory and pro-thrombotic state (63).

The MedDiet may help mitigate these risks by supporting a healthier lipid profile-reducing total cholesterol, low-density lipoprotein cholesterol, triglycerides, oxidized low-density lipoprotein and apolipoprotein B, while increasing high-density lipoprotein cholesterol and apolipoprotein A1. It may also contribute to a more favorable fatty acid intake, characterized by higher consumption of monounsaturated and polyunsaturated fatty acids and lower intake of saturated fatty acids. This can help reduce inflammatory burden and the risk of atherosclerosis, particularly in individuals with familial hypercholesterolemia and non-alcoholic fatty liver disease (45, 51, 59). Similarly, the MedDiet improves glucose metabolism, lowering fasting glucose, insulin and HbA1c (45, 60), while enhancing energy and amino acid metabolite profiles, reflecting improved metabolic flexibility and mitochondrial function (42, 51).

### 2.3 Integrated biomarker overview

Taken together, 142 distinct proteins and metabolites were identified (Supplementary Table S4), spanning a broad range of biological categories, including vitamins and antioxidants, fatty acids and lipid metabolism, energy-related metabolites, chemokines, cytokines, growth factors, receptor proteins, vascular adhesion molecules, endocannabinoids and gut-derived or xenobiotic metabolites. These findings highlight multiple interconnected pathways through which the MedDiet may modulate inflammation, oxidative stress, and vascular function, i.e., contributing to protection against aging-related diseases. However, the frequency with which these biomarkers were reported across the 95 studies varied considerably (Table 4). This reflects both intrinsic heterogeneity in study designs, populations, and analytical approaches, as well as limited cross-study replication of individual biomarkers. Together, these factors pose important constraints for data comparability and reuse across studies and reinforce the need for standardized reporting and FAIR-compliant data practices to enhance interoperability, and secondary data use.

**Table 4:** Plasma biomarkers reported in ≥3 research articles from the 95 selected publications.

Number of publications	Other biomarkers
34	Cholesterol^**^
13	Glucose
12	Triglycerides^*^
11	Carotenoids^****^
9	Aminoacids^***^
7	HbA1c^*a*^/MDA
6	DHA (omega-3 FA)
5	EPA (PUFA)/SFA
4	MUFA
3	ALA (omega-3 FA)/LA (linoleic acid, PUFA)/vitamin E

Supporting information available as Supplementary Table S4.

^*a*^HbA1c reflects the non-enzymatic glycation of hemoglobin that occurs under chronically elevated glucose conditions.

^*^Triglycerides: triglycerides are key biomarkers, where elevated levels indicate increased risk of cardiovascular disease, atherosclerosis, and pancreatitis. They are classified as saturated or unsaturated.

^**^Cholesterol: high-density (HDL) and low-density lipoprotein (LDL) levels indicate the amount of circulating cholesterol, a lipid produced

endogenously and essential for cell membranes, hormone synthesis, and vitamin D production.

^***^Aminoacids: amino acids such as homocysteine, betaine, alanine, glutamine, lysine, 3-methylhistidine, L-serine, and branched-chain amino acids (BCAAs) are fundamental for protein synthesis, supporting muscle growth and repair, hormone and neurotransmitter production, and immune function.

^****^Carotenoids: lycopene, lutein, zeaxanthin, carotenediol, and β-carotene serve as carotenoid biomarkers of intake, status, and biological activity.

At the metabolite level, the frequent reporting of cholesterol, triglycerides, glucose, HbA1c, carotenoids and MDA reflect MedDiet modulation of metabolic risk factors for cardiovascular disease and diabetes. Cholesterol and triglycerides are well-established lipid markers of cardiovascular risk, while glucose and HbA1c are glycemic markers (60). Carotenoids, as dietary antioxidants, and MDA, a marker of lipid peroxidation, illustrate the MedDiet effects on antioxidant status and the regulation of oxidative stress (67–70).

At the protein level, several proteins were consistently reported across the 95 research articles considered. In Table 5, proteins reported in ≥ 5 publications were included. The most frequently reported proteins (≥10 publications) were CRP and the cytokines IL6 and TNF, which are well established markers of inflammation and immune response activation. Additional inflammatory mediators, including CCL2 and MPO, are involved in leukocyte recruitment and oxidative stress (63). LEP, APOB, APOA and INS are proteins related to metabolic homeostasis, lipid transport, insulin signaling, adipokine regulation, and cardiometabolic disease pathways (34, 58). The less frequently reported proteins may represent emerging candidates. Nevertheless, and independently of the reported frequency, further validation is required in independent cohorts and longitudinal studies to confirm their robustness and biological relevance.

**Table 5:** Plasma biomarkers reported in ≥5 research articles from the 95 selected publications.

Number of publications	Plasma protein biomarkers (Gene name)	Plasma protein biomarkers (Protein name)	Associated conditions	Biological interpretation
24	*CRP*	C-reactive protein (CRP)	Inflammation, CVD, atherosclerosis, insulin resistance, obesity, NAFLD, Alzheimer's	↑= adverse inflammation; ↓= beneficial
18	*IL6*	Interleukin-6 (IL-6)	Inflammation, CVD, atherosclerosis, T2D, aging, NAFLD	↑= pro-inflammatory; ↓= beneficial
12	*TNF*	Tumor necrosis factor alpha (TNF-α)	Inflammation, CVD, T2D, cancer, obesity, aging, neurodegeneration	↑= pro-inflammatory; ↓= beneficial
8	*LEP*	Leptin	CVD, Insulin resistance, T2D, obesity, NAFLD, inflammation	↑= obesity/leptin resistance; ↓= improved metabolic status
7	*APOB*	Apolipoprotein B (ApoB)	Atherosclerosis, CVD, CHD, inflammation, obesity/T2D, aging	↑= atherogenic; ↓= beneficial
6	*APOA/INS*	Apolipoprotein A (ApoA1)/insulin	T2D, CVD, CHD/T2D, obesity, CHD, metabolic syndrome	↑= cardioprotective; ↑= insulin resistance; ↓= improved insulin sensitivity
5	*CCL2/MPO*	C-C motif chemokine ligand 2 (MCP-1)/myeloperoxidase (MPO)	T2D, CVD, atherosclerosis/NAFLD, inflammation	↑= oxidative stress; ↓= beneficial

Supporting information available as Supplementary Table S4. Legend of the associated conditions: T2D, type 2 diabetes; CVD, cardiovascular disease; CHD, coronary heart disease, NAFLD, non-alcoholic fatty liver disease; MAFLD, metabolic dysfunction-associated fatty liver disease.

Given the consistent reporting of the protein biomarkers from Table 5, and since proteins rarely function in isolation, we next explored their interactions within a protein-protein interaction network to assess whether this approach could provide additional insight into the molecular mechanisms by which the MedDiet may exert anti-inflammatory, vascular-protective and metabolic effects. As proteins act as enzymes, receptors, structural components and signaling mediators, their abundance and activity can reflect processes at both cellular and systemic levels, making them valuable indicators for linking MedDiet adherence to clinically meaningful outcomes (42).

## 3 Plasma protein biomarkers network

The protein-protein interaction network was generated using the STRING database, integrating known and predicted functional associations derived from curated databases, experimental evidence, co-expression, text mining, and genomic context (71). In this updated analysis, only plasma protein biomarkers reported in five or more independent studies were included, to focus on the most consistently replicated findings and improve the robustness and reliability of the network. A high-confidence interaction score was applied to minimize weak or potentially spurious associations and to retain the most biologically relevant connections.

**Figure 2:**
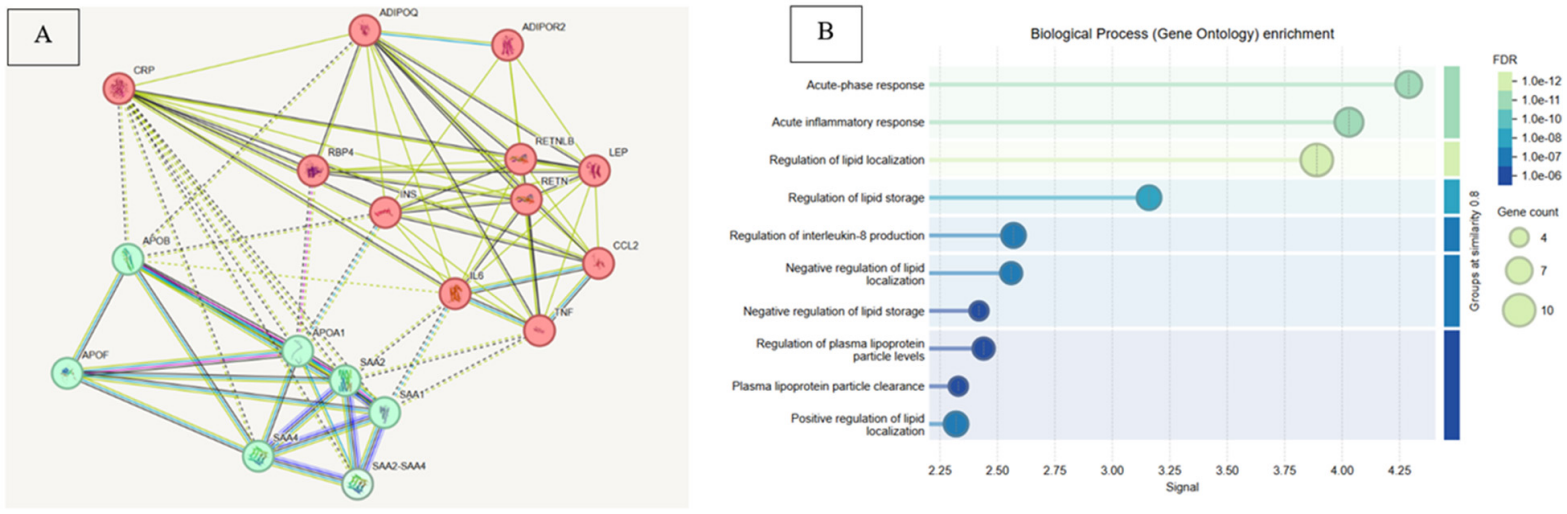
Network and functional enrichment analysis of plasma protein biomarkers. **(A)** Protein-protein interaction network. Nodes represent proteins and edges represent known or predicted associations derived from curated databases, experimental evidence, co-expression, text mining, and protein homology. The network demonstrates two interconnected functional modules related to inflammatory signaling and lipoprotein metabolism, linked by central proteins. **Protein abbreviations:** ADIPOQ, adiponectin; ADIPOR2, adiponectin receptor 2; APOA1, apolipoprotein A-I; APOB, apolipoprotein B; APOF, apolipoprotein F; CCL2, C-C motif chemokine ligand 2; CRP, C-reactive protein; IL6, interleukin-6; INS, insulin; LEP, leptin; RBP4, retinol-binding protein 4; RETN, resistin; RETNLB, resistin-like beta; SAA1, serum amyloid A1; SAA2, serum amyloid A2; SAA4, serum amyloid A4; TNF, tumor necrosis factor. **(B)** Gene Ontology Biological Process enrichment analysis of the same protein set. Bubble size reflects gene count, color indicates the false discovery rate (FDR), and the x-axis represents enrichment signal strength.

The resulting network revealed a tightly connected structure centered on biomarkers related to inflammation, endothelial and cardiovascular function, and lipid and glucose metabolism (Figure 2A). Two major functional clusters were apparent. The first cluster was enriched for inflammatory and acute-phase proteins, including CRP, IL6, TNF, LEP, resistin (RETN), resistin-like beta (RETNLB) and CCL2, indicating coordinated regulation of innate immune activation, cytokine signaling, and adipose tissue-derived inflammatory pathways. The second cluster was mainly composed of proteins involved in lipoprotein metabolism and transport, including APOA1, APOF, APOE and serum amyloid A (SAA1, SAA2 and SAA4), suggesting close links between lipid handling, reverse cholesterol transport, and inflammatory remodeling of lipoproteins (72).

Notably, APOA1 occupied a central bridging position between the inflammatory and lipid-related modules, supporting its recognized role as both a structural component of high-density lipoprotein and a mediator of anti-inflammatory and antioxidant functions (44). These findings highlight a strong integration between metabolic and immune pathways, consistent with the concept of immune regulation underlying MedDiet-associated health benefits (58).

Gene Ontology enrichment analysis further supported these observations (Figure 2B). The most significantly enriched biological processes included acute-phase response, acute inflammatory response, regulation of lipid localization, regulation of lipid storage and regulation of interleukin-8 production. Additional enrichment for plasma lipoprotein particle clearance and positive regulation of lipid localization further emphasized the central role of lipid trafficking pathways. Collectively, these results suggest that the most consistently reported plasma biomarkers linked to MedDiet adherence converge on pathways involved in systemic inflammation control, lipid transport, and cardiometabolic homeostasis (73).

**Figure 3:**
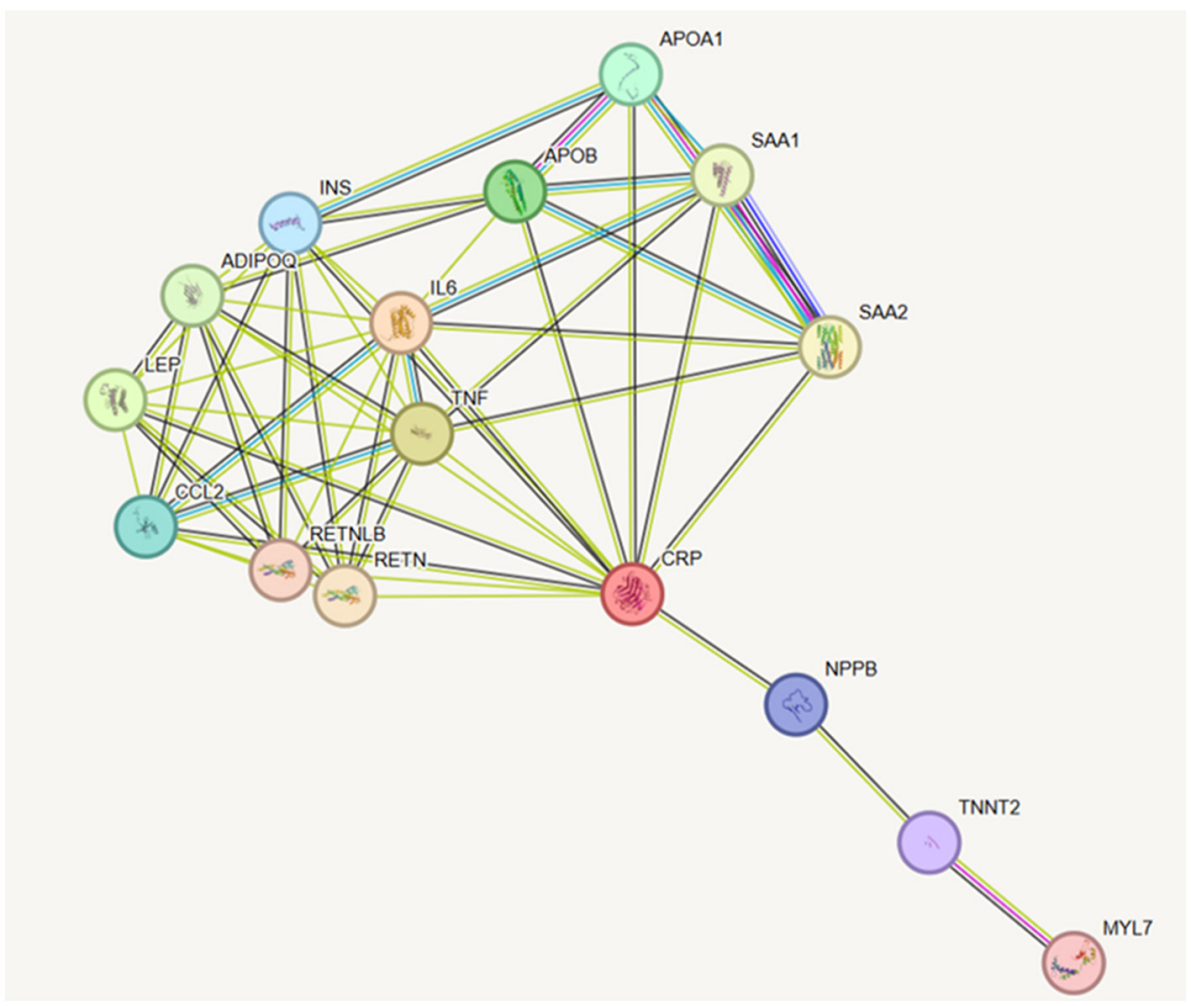
Protein-protein interaction network integrating aging-associated cardiovascular proteins with plasma biomarkers related to inflammation, endothelial and cardiovascular function, and lipid and glucose metabolism. Nodes represent proteins and edges represent known or predicted associations derived from curated databases, experimental evidence, co-expression, text mining, and protein homology. Protein abbreviations: ADIPOQ, adiponectin; APOA1, apolipoprotein A-I; APOB, apolipoprotein B; CCL2, C-C motif chemokine ligand 2; CRP, C-reactive protein; IL6, interleukin-6; INS, insulin; LEP, leptin; MYL7, myosin light chain 7; NPPB, natriuretic peptide B (B-type natriuretic peptide); RETN, resistin; RETNLB, resistin-like beta; SAA1, serum amyloid A1; SAA2, serum amyloid A2; TNF, tumor necrosis factor; TNNT2, troponin T2, cardiac type.

Given growing evidence that chronic low-grade inflammation is a major contributor to accelerated biological aging and age-related cardiovascular decline (73), we further explored whether protein–protein interaction analysis could identify molecular links between inflammatory/metabolic biomarkers and proteins associated with cardiac aging. For this purpose, a new STRING network was generated using the previously selected plasma biomarkers, together with established aging-related cardiovascular proteins natriuretic peptide B (NPPB), troponin T2 cardiac type (TNNT2), and myosin light chain 7 (MYL7) (Figure 3). The network suggested that cardiovascular aging biomarkers displayed fewer direct mechanistic connections with the core inflammatory-metabolic cluster. Notably, NPPB was mainly connected through CRP, a broad systemic inflammatory marker, which is consistent with the non-specific inflammatory and age-associated nature of both biomarkers rather than indicating a dedicated functional axis. Similarly, TNNT2 and MYL7, markers associated with cardiomyocyte injury and contractile remodeling, extended downstream through sparse associations pre-dominantly supported by literature- and expression-based evidence, suggesting weaker functional integration within the central inflammatory-metabolic network (74). The position of CRP as an interface node between the main inflammatory network and the cardiac aging cluster is particularly noteworthy. As the most consistently reported circulating biomarker in our systematic review, CRP may represent a molecular bridge linking chronic inflammation with progressive myocardial dysfunction. This finding is consistent with evidences associating persistent low-grade inflammation with fibrosis, ventricular remodeling, endothelial dysfunction, and subsequent heart failure development (74). Overall, the network confirms as expected that cardiovascular aging may be biologically connected to systemic inflammatory and metabolic dysregulation, but the evidence for the integrative circulating markers of myocardial injury with upstream immune-metabolic pathways are currently limited (74). This partial separation may also reflect differences in biomarker reporting frequency, tissue specificity and varying sensitivity of current proteomic studies. Future investigations using harmonized longitudinal biomarker panels and multi-omics integration will be important to better define the transition from inflammaging to overt cardiac dysfunction.

## 4 Conclusion

The present review supports the view that the MedDiet modulates a coordinated immune-metabolism network involving inflammatory mediators, lipid transport proteins, metabolic regulators, and circulating markers of cardiovascular function. Across the integrated analyses, the most consistently reported biomarkers converged on key inflammatory mediators, particularly IL6, TNF, and CRP, together with proteins involved in lipid metabolism and metabolic regulation, including APOA1, APOB, ADIPOQ, INS and LEP. These findings suggest that, rather than acting through isolated molecular targets, the MedDiet its beneficial effects by modulating interconnected immune, metabolic, and vascular pathways relevant to cardiometabolic health.

Integration of aging-associated cardiovascular biomarkers into the protein–protein interaction network indicated that markers of myocardial stress and injury, such as NPPB and MYL7, were only partially connected to the core inflammatory-metabolic cluster. The bridging position of CRP suggests that chronic low-grade inflammation may represents an important molecular interface between systemic metabolic dysregulation and progressive cardiovascular aging. Although these findings support the concept that inflammaging contributes to the transition from metabolic dysfunction to cardiac impairment, further experimental and longitudinal studies are required to validate these relationships.

Despite these insights, this review has several limitations. Heterogeneity in biomarker reporting, analytical platforms, study design, and the limited standardization of methodologies reduce comparability across studies and constrain network resolution. Moreover, the frequency with which a biomarker is reported does not necessarily reflect its biological importance, and potentially relevant proteins may remain underrepresented in literature. Future studies integrating harmonized proteomic and multi-omics datasets, together with longitudinal and intervention-based designs, will be essential to refine the mechanistic understanding of MedDiet-related metabolic regulation. Further research should also systematically incorporate other components of the traditional Mediterranean lifestyle, including physical activity, social interactions, sleep patterns and food preparation practices, to provide a more comprehensive systems-level understanding of its effects on healthy aging and cardiometabolic health.
